# The patterning toolbox FIB-o-mat: Exploiting the full potential of focused helium ions for nanofabrication

**DOI:** 10.3762/bjnano.12.25

**Published:** 2021-04-06

**Authors:** Victor Deinhart, Lisa-Marie Kern, Jan N Kirchhof, Sabrina Juergensen, Joris Sturm, Enno Krauss, Thorsten Feichtner, Sviatoslav Kovalchuk, Michael Schneider, Dieter Engel, Bastian Pfau, Bert Hecht, Kirill I Bolotin, Stephanie Reich, Katja Höflich

**Affiliations:** 1 Ferdinand-Braun-Institut gGmbH, Leibniz-Institut für Höchstfrequenztechnik, Gustav-Kirchhoff-Str. 4, 12489 Berlin, Germany; 2 Corelab Correlative Microscopy and Spectroscopy, Helmholtz-Zentrum Berlin für Materialien und Energie, Hahn-Meitner-Platz 1, 14109 Berlin, Germany; 3 Max Born Institute for Nonlinear Optics and Short Pulse Spectroscopy, Max-Born-Straße 2A, 12489 Berlin, Germany; 4 Freie Universität Berlin, Arnimallee 14, 14195 Berlin, Germany; 5 Department of Experimental Physics 5, Röntgen Research Center for Complex Material Research (RCCM), Physics Institute, University of Würzburg, Am Hubland, D-97074 Würzburg, Germany; 6 Dipartimento di Fisica, Politecnico di Milano, Piazza Leonardo da Vinci, 32 20133 Milano, Italy

**Keywords:** automated patterning, focused He ion beam, graphene, magnetic multilayers, mechanical resonator, pattern generation, plasmonic antennas, two-dimensional materials

## Abstract

Focused beams of helium ions are a powerful tool for high-fidelity machining with spatial precision below 5 nm. Achieving such a high patterning precision over large areas and for different materials in a reproducible manner, however, is not trivial. Here, we introduce the Python toolbox FIB-o-mat for automated pattern creation and optimization, providing full flexibility to accomplish demanding patterning tasks. FIB-o-mat offers high-level pattern creation, enabling high-fidelity large-area patterning and systematic variations in geometry and raster settings. It also offers low-level beam path creation, providing full control over the beam movement and including sophisticated optimization tools. Three applications showcasing the potential of He ion beam nanofabrication for two-dimensional material systems and devices using FIB-o-mat are presented.

## Introduction

Future breakthroughs in nanotechnology will rely on the ability to fabricate materials and devices by design, that is, to tailor both material properties and device geometries according to a sophisticated blueprint. Thin layers and two-dimensional (2D) materials are especially interesting candidates for designer materials [1] as they are compatible with planar device geometries and may be combined in a straightforward manner, for example, by stacking. Applications of such materials may rely on (coupled) material excitations, such as plasmon polaritons in gold nanostructures [2], on physical properties, such as the exceptionally high mechanical stability of suspended graphene [3], or on asymmetric magnetic interactions as in Co/Pt films, enabling the formation of desired spin textures [4]. As the actual device geometry determines the response to external stimuli, the coupling strengths, and the corresponding figures of merit, ultimate control in nanopatterning down to the single-digit nanometer range is heavily sought after.

One promising candidate for ultraprecise nanofabrication is focused ion beam (FIB) machining. Focused ion beams locally remove material based on physical sputtering with a large degree of flexibility due to advanced beam control. FIB patterning is a direct single-step process without the need of potential contaminants, such as the resists used in lithographic approaches. For conventional gallium (Ga) ion beams the achievable minimum feature sizes are still limited to approx. 10 nm [2], and Ga implantation may cause unwanted modification of material properties. In contrast, the recently emerged helium ion microscopes (HIM) provide beam spot sizes below 1 nm [5]. The beam is formed by ionizing helium (He) atoms at an atomically sharp tip consisting of three tungsten atoms (trimer). The trimer is metastable and has to be reformed at irregular time intervals ranging from several days to one or two months. For imaging and nanofabrication, only one of the three atoms is selected. This nearly ideal point source allows not only for high-resolution imaging but also for the milling of smallest geometric features [5–7]. Furthermore, large-area machining is possible due to the extraordinary beam stability in combination with a large depth of focus. The first implies that the focus quality remains unchanged over long time intervals, for example, overnight, while the latter ensures that sample geometry and the slight unavoidable tilt of the sample does not reduce patterning quality. Hence, interim imaging and, thus, unintended ion beam modification can be avoided.

The FIB patterning process relies on the beam control that is well advanced and readily available in commercial ion microscopes. Ion microscopes of all manufacturers are equipped with patterning engines, that is, a digital-to-analog converter (DAC) patterning board, defining pixels that can be addressed by the beam, and a corresponding software that allows the user to set the beam parameters and to create geometries with specific raster styles. However, such vendor-specific software is adapted to standard use cases and lacks the flexibility to realize ultimate fidelity and robust large-area machining. So far, several patterning tools were developed, some of them only for specific photonic components, such as solid immersion lenses from diamond [8] or hole arrays and groove waveguides [9]. Others are more versatile but require proprietary software or are non-public. Hence, they are of restricted use for the community. Interesting approaches for the patterning of three-dimensional surface profiles rely on the creation of the respective three-dimensional objects in computer-aided design (CAD) software [10–14]. Here, the corresponding CAD file is converted to more general file types, such as stereo lithographic files (.stl) [10] and G-code [14], or directly into files that encode beam positions, so-called stream files [12]. In all these cases, it is beneficial to perform the material removal in thin slices of equal dose instead of relying on locally varying doses of a single slice [11]. This is a great benefit over the manufacturer-specific patterning options that allow for grey-scale patterning, where the grey values encode local doses. The results of the patterning can be improved through modeling of the relevant processes in FIB machining, especially angle-dependent physical sputtering [11] and redeposition [15], or geometric considerations [12]. In the same manner, locally varying doses in He ion-based resist patterning may be corrected based on heuristic modeling employing a point spread function that sums up all physical and chemical processes in resist activation [16]. All these methods have in common that the control over the actual beam path is limited. While there are attempts to reduce the amount of blanking operations [13] and to follow the geometry of the pattern, tools that allow one to create arbitrary patterns with a geometry-adapted beam path are not yet available. Since the beam path is of utmost importance for the patterning result, new tools are needed to achieve both ultimate resolution and shape fidelity [17].

Here, we introduce the modular patterning toolbox FIB-o-mat to create and optimize patterns for ion beam machining, including automation [18]. The idea of FIB-o-mat is to make use of the built-in functionality of the commercial patterning engine where appropriate, but to provide advanced options where required. FIB-o-mat provides an easily extendable modular toolbox to enable full control over the beam movement. The user can design patterns and subsequently specify optimized beam paths, which are translated into a file format appropriate for the respective patterning engine. Here, the capabilities and first implementations are demonstrated for He ion beam patterning in three different use cases. In general, FIB-o-mat is usable for all sorts of ions and microscopes of multiple manufacturers. Only the output files have to be adapted to the specific patterning back end.

## Ion Beam Machining with Light Ions

Focused Ga ion beams are ubiquitous in ion beam machining with well-established applications in material characterization, for example, TEM lamella fabrication, cross sections or tomographies [19–20], or in the fabrication of prototype nanostructures, such as plasmonic antennas [2]. In contrast, appropriate fields of application for focused beams of light ions are still under exploration. This is a consequence of the novelty of the technique and of several significant peculiarities in the behaviour of light ions upon interaction with a solid.

First, light ions exhibit a large interaction volume in the solid. The penetration depth of 30 keV He ions in silicon is more than five times larger than the penetration depth of Ga ions of the same energy [21]. The consequently large collision cascade may create a significant amount of heat. Even for small ion doses deformation of the manufactured structures can be observed when placed on a material of small heat capacity, such as glass (cf. subsection 3 of “Results and Discussion”). Here, it has to be mentioned that separating the influence of local heat creation from other beam-induced effects is not trivial (see Supporting Information File 1 for further details). Another consequence is the deep implantation of light ions. He and Ne are chemically inert and may therefore diffuse out of the substrate after a while. Diffusion in solids, however, is extremely inefficient, such that the majority of the primary ions will be implanted at their final trajectory position [22]. Hence, large ion doses lead to gas agglomeration and the formation of bubbles, manifesting as strong surface swelling [23]. Furthermore, the associated sputter rate of light ions is roughly an order of magnitude smaller than that of Ga ions [21–22].

In addition, the low ion mass has further implications. The local ion–solid interaction is a balance among several ion-induced, surface-related, and thermally triggered processes [24]. Physical sputtering is only one of the processes. Also, chemical reactions with adsorbed contaminants can occur and, under certain circumstances, may dominate over the atomic knock-out. Typically, ion beam machining is carried out under high-vacuum conditions, where the amount of contaminants in the chamber (mostly water, but also hydrocarbons) still can form a monolayer per second on average. Often, additional contaminants may be present on the sample surface, for example, residues from wet-chemical processing. The locally introduced energy can mobilize these contaminants such that they diffuse towards the beam center where they are polymerized [24]. In the worst case, this results in material build-up instead of sputtering. Already minor surface contamination can lead to unwanted side effects, such as carbonized edges that may change local material properties. Furthermore, locally varying sputter rates and increased minimum doses for sufficient material removal and increased heat damage may occur. All samples that are inert under oxidizing atmosphere should be cleaned in an oxygen plasma before ion beam machining. Generally, all samples should be introduced into the vacuum system one day in advance to allow possible contaminants to desorb from the samples and be pumped out. While these protocols can improve the technical conditions for the machining with light ions, other challenges such as small sputter rates and large interaction volumes persist.

Therefore, 2D materials are an ideal platform for ion beam machining with light ions. The lack (or non-relevance) of the interaction volume allows for a high spatial resolution, enabling the fabrication of structural features in the single-digit nanometer range where small sputter rates play a minor role. This holds true not only for monolayer 2D materials, such as graphene, but also for thin films forming quasi 2D geometries. The versatility of the corresponding materials opens a wide field of exciting applications including, but not limited to, the direct writing of defects to act as nuclei for epitaxial growth [25], the fabrication of two-dimensional phononic crystals [26], the magnetic patterning of suspended Co/Pt multilayers, the fabrication of two-dimensional mechanical resonators based on single-layer graphene, and the fabrication of coupled plasmonic nanoantennas from single-crystalline gold. The three latter examples are realized in this work by developing optimized patterning and automation routines [18].

## Patterning and Beam Control

**Figure 1:**
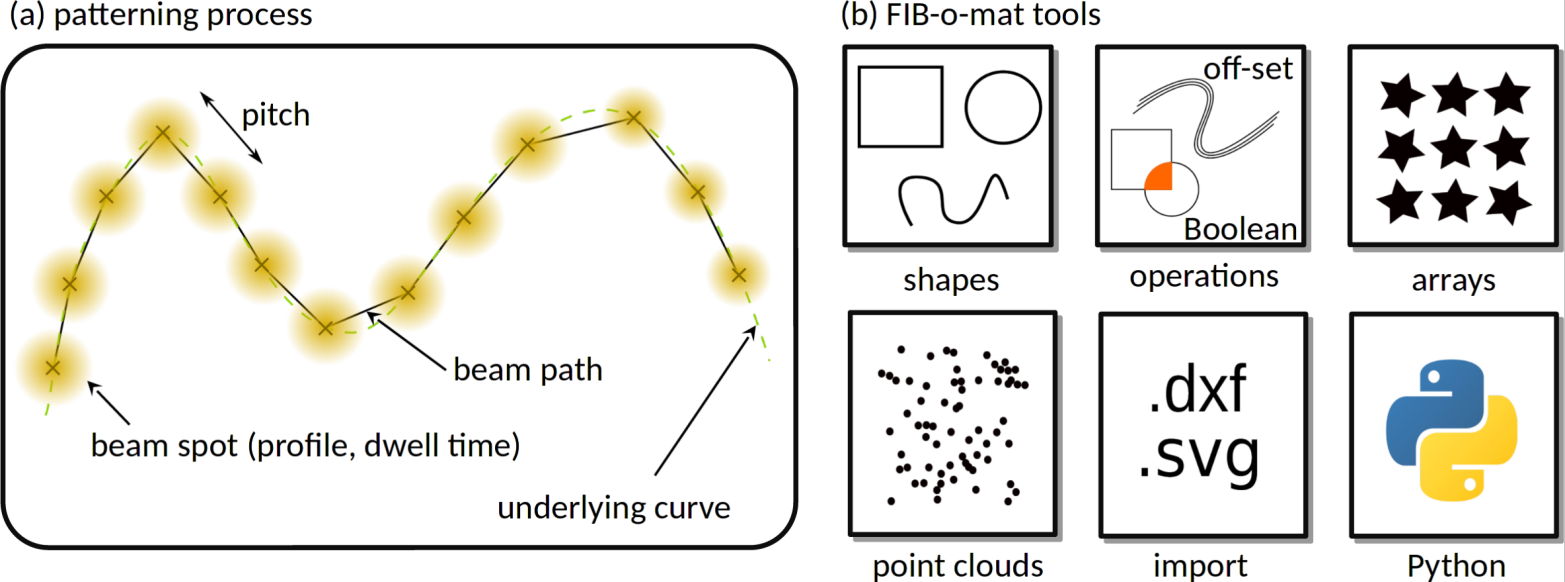
FIB-o-mat overview. (a) For patterning the beam spot follows a rasterized beam path with a defined pitch and dwell time per spot. The piece-wise continuous beam path could be generated from a curved shape (lime curve). Positions of the spots are given by the kinks of the beam path. (b) To create such beam paths FIB-o-mat provides geometric primitives that can be combined by Boolean operators, arranged in lattices and rasterized in different styles using the high-level beam path generation available from the microscope patterning software. Since high-fidelity patterning requires the adaption of the beam path to all edges of the geometry, the low-level beam path generation allows for the creation of point clouds in various ways, for example, by curve off-setting either of geometric primitives or imported graphics. Additionally, all Python functionality is available in FIB-o-mat.

Patterning with an ion beam is a digital process where the beam spot dwells for a defined time at a fixed location and is then displaced by a defined distance (‘pitch’) to dwell again. To avoid unintended beam damage the ion beam may by blanked when it is displaced over larger distances. Figure 1a depicts a beam path, which is defined by the spot locations, their dwell times, and their pitches. The beam profile depends on the beam settings, given by acceleration voltage, extractor voltage (named ‘best imaging voltage’ (BIV) in the case of He ion microscopy), and the beam current, as well as on the quality of the focus. The beam profile is unknown a priori but can be typically described with a Gaussian profile in He ion microscopy [27]. It has to be mentioned that the beam parameters for a gas field ion source (GFIS) are strongly correlated and, thus, not independently adjustable. Due to the extremely small size of the source typical currents are only in the range of picoamperes. Varying the gas flux can finely adjust the beam current. However, too large gas fluxes lead to a reduced trimer lifetime. Otherwise, the current can be modified by the size of the selected aperture and by the location of the beam crossover relative to this aperture (named ‘spot control’ by the manufacturer). Placing the crossover into the aperture leads to the largest possible current but also to the most divergent beam. Placing the crossover above the aperture reduces not only the beam current but also all image errors by cutting off non-paraxial radiation. While the local sputter rate depends strongly on the primary ion flux and, therefore, the beam current, the fidelity of the structures requires an optimum beam profile. Hence, in patterning, typically, a compromise between the optimum beam properties and the largest possible sputter rate has to be found. Usually, the beam path is inferred from the priorly optimized beam settings and the specific target geometry. In the following, the combination of beam settings and patterning geometry is called ‘pattern’.

Depending on the target geometries, the pattern creation process can become a complicated and potentially time-consuming task if done by hand. An automated and computer-aided patterning design process can simplify this task tremendously. In case of large arrays, even if the individual shapes are not complex themselves, the large number of them makes it unreasonable to create and layout everything manually. Also, if a pattern has to match desired geometric constraints, an automated pattern generation process can construct the complete pattern from few user-provided parameters by exploiting the defined constraints. This provides easy access to systematic variation of geometries and raster strategies. Finally, for complex pattern geometries, especially with curved edges, the available features of commercial patterning control software are not sufficient to even create the corresponding adapted beam paths. To address these issues, we developed the pattern generation toolbox FIB-o-mat with a Python interface. FIB-o-mat enables the creation of arbitrarily shaped pattern geometries in combination with geometry-adapted beam paths and optimization/automation tools. The code and Python package documentation can be found online at gitlab under the gpl3 license [18,28–29]. Pre-build packages are available on pypi [30].

## The FIB-o-mat Toolbox

The overall design of FIB-o-mat relies on a two-step pattern definition: First, a pattern shape must be defined and, second, appropriate beam settings can be added to the pattern shape. By design, neither assumptions are made on certain settings (for example beam profile) nor are default values used. This may limit the convenience for standard tasks compared to vendor-specific patterning software but, in return, the design process is completely transparent to the user.

Figure 1b depitcs schematically the available tools to provide a maximum flexibility for pattern generation. To generate shapes, FIB-o-mat provides a range of geometric primitives, including points, polygons and lines, ellipses, arc splines, and parametric curves. These can be used as building blocks for custom shapes. Alternatively, .svg and .dxf files can be imported and their enclosed geometries will be automatically translated to geometric primitives defined in FIB-o-mat. All shapes can be combined via Boolean operations and arranged in arbitrary two-dimensional lattices by defining a unit cell and displacement vectors. This is accomplished without explicit rasterization and, consequently, the process again results in analytic shape geometries. For rasterization of the obtained geometries, different line-by-line raster styles are available, such as serpentines or cross sections. Further, shapes can be off-set along their normal direction. If provided by the manufacturer, such curves may be loaded into the patterning engine and rasterized there. In most cases they are used to generate point clouds in which each point represents a beam location. This provides the possibility to adapt the beam path to arbitrary curved geometries, not only for the built-in primitive shapes and the Boolean derivatives but also for arbitrary imported shapes. Finally, full Python functionality is available with FIB-o-mat, for example to employ other geometric modeling libraries.

Pattern generation can be carried out at two different abstraction levels, depending on the needs of the user and the capabilities of the microscope software. High-level beam path generation is based on geometric primitives and raster routines available in the patterning generator of the microscope. Low-level beam path generation creates point clouds, which directly define the ion beam path.

### 1 High-level beam path generation and automation

For the high-level approach, the pattern is constructed from geometric primitives available in the patterning software of the microscope. In the same manner, the rasterization relies on the available parameters such as pitch, line distance, and line ordering in a rectangular pattern. The parameters have to be specified by the user within FIB-o-mat, which, in turn, generates a pattern file containing the shapes and the patterning parameters. The actual rasterizing of these geometric primitives is carried out by the patterning engine of the microscope. If, for example, a rectangle shall be rasterized line-by-line but with varying pitches for each line, FIB-o-mat would generate a list of lines with the corresponding raster parameters defined for each line.

**Figure 2:**
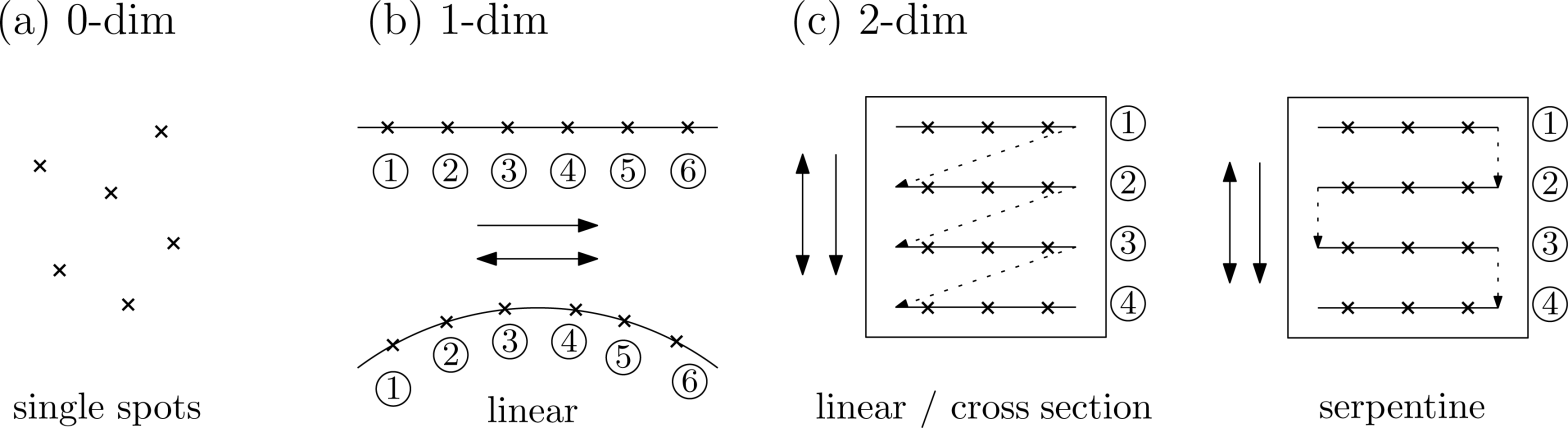
Geometric primitives and rasterization styles available for high-level pattern generation. (a) Single spots, (b) straight lines and circle outline segments, and (c) a rectangle as representative for available 2D primitives.

The rasterization process for the high-level approach is schematically depicted in Figure 2. The simplest geometric entity is a spot. As shown in Figure 2a, spots with well-defined positions and corresponding dwell times and number of repeats can be created by FIB-o-mat. Typically, the only one-dimensional geometries available in commercial patterning software are straight lines and circle segments and the rasterization has to be carried out in a consecutive way, cf. Figure 2b. In the same manner, all two-dimensional geometric primitives have to make use of the pre-defined raster styles, such as line-by-line in one direction or alternating directions as well as cross-sectional rasterization (repeat each line until the full dose is applied before starting the next line) or serpentines (make the beam path continuous by turning the direction when starting the next line). In the case of circular primitives, rasterization in an azimuthal direction (raster lines follow the circular outline of the shape) is implemented by all major manufacturers.

There are several benefits of the high-level approach for patterning. As it relies on predefined geometric entities, the loading of generated patterning files can be faster than that of potentially huge point clouds. Especially a collection of many simple patterns with changing geometry or rasterization parameters will still result in relatively small file sizes. In addition, the patterning settings of the individual objects can be easily changed in the patterning software after loading the pattern file created with FIB-o-mat. While other microscopy parameters, such as the stage position, can be controlled via an application programming interface (API) or patterning options of the manufacturer, large-area patterning can be automated by FIB-o-mat. Another interesting approach for the automation of small and regularly arranged patterns makes use of the pitch in high-level patterns [31]. When setting the pitch of a 10 μm rectangle to 10 nm, an array of 1000 × 1000 holes with 10 nm spacing may be created. By placing such rectangles onto a rasterized curve, this curve will be replicated in the same manner. While this approach is fast and efficient for large arrays of patterns consisting of few points, it becomes unreasonable for arranging more complex subunits.

However, the flexibility of the high-level approach is low. Only geometric primitives pre-defined by the manufacturer along with few raster styles are available. For complex shapes, the available raster routines even reduce to a line-by-line rectangular raster once different geometric primitives are combined by Boolean operations. Therefore, in many application cases the beam path will not follow the geometry edges, leading to artifacts, especially when approaching the resolution limit of the machine.

Attention has to be paid when employing the geometric primitive ‘spot’ of the manufacturer software. The commercially implemented patterns contain not only the geometric shapes and raster styles but additional optimization strategies that are often not transparent to the user. One example is a beam stabilization position along with a certain waiting time after each repeat of the pattern. What may have a minor influence for two-dimensional patterns can become a major issue in the case of point clouds. An estimate for one of the established manufacturers gave a beam stabilization time of about 5 ms for each pattern summing up to almost 1.5 h when executing 1 million spots. Given the fact that typical dwell times are in the range of microseconds, the actual patterning time of this point cloud would be expected to take seconds.

### 2 Low-level beam path generation and optimization

**Figure 3:**
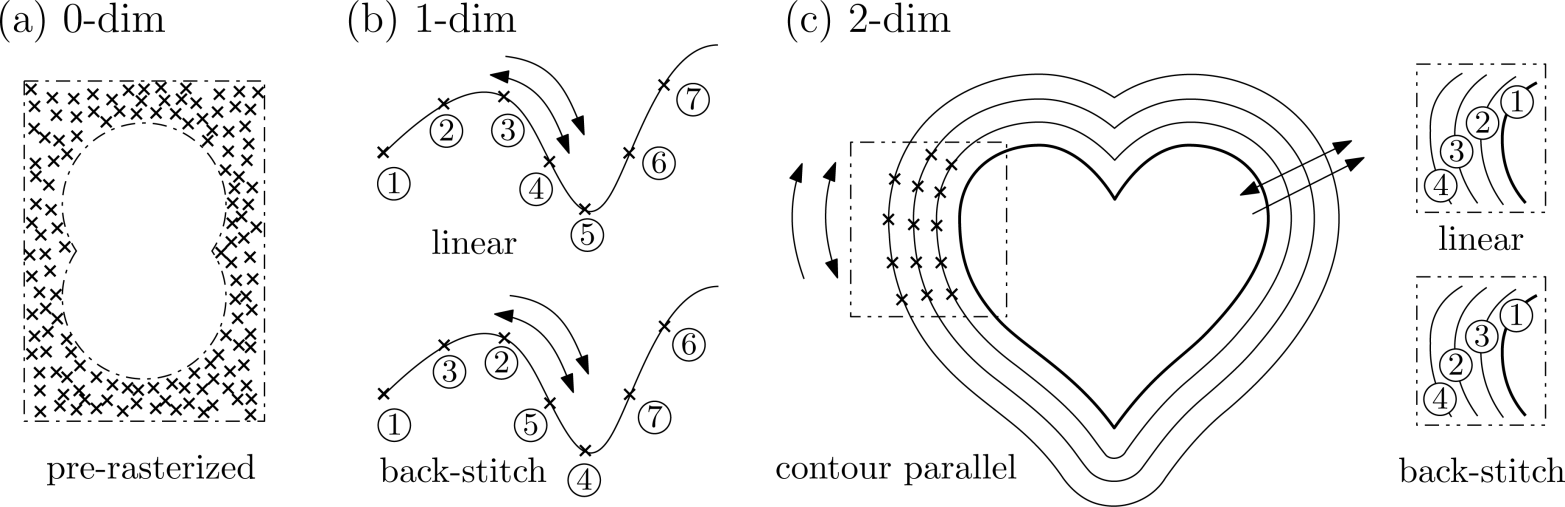
Geometric entities and rasterization styles available for low-level pattern generation. (a) Pre-rasterized point cloud, (b) arbitrary parametrized curves with custom rasterization, and (c) a heart as representative for arbitrary 2D geometries that can be either generated or loaded including custom raster styles.

Full control over the beam path is only provided by the low-level tools of FIB-o-mat. Here, the point cloud depicted in Figure 3a is not an assembly of pre-defined primitives. Instead, it represents a list of true beam positions. Such lists can be loaded into the microscope software as so-called deflection lists or stream files. FIB-o-mat creates the deflection lists by defining individual points as pre-rasterized geometry, as shown in Figure 3a, by rasterizing arbitrary parametrized curves, or by calculating off-set curves around arbitrary geometries as displayed in Figure 3b,c. In the latter two cases the rasterization of the curves can be defined in a custom manner. This includes all raster styles that are available in the high-level approach but also user-defined styles, such as the back-stitching depicted in Figure 3b,c. The provided tools allow for flexible pattern generation and mitigate typical problems in ion beam-based machining.

As the local material removal by ion beam impact is a complex interplay between different physical and chemical processes, many unwanted artifacts can (and typically will) occur during patterning [24]. The ion beam-induced physical sputtering does not cause a complete material removal. Instead, the material is locally redistributed [15]. The phenomenon is known as redeposition and can be minimized by varying the slow beam direction [17], that is, for line-by-line scanning, by starting from the first line in the first repeat and from the last line in the second repeat.

During ion beam machining of multi-crystalline materials the varying crystal orientations cause a variation in the local sputter rate due to ion channeling [24]. If one of the symmetry axes of the crystal lattice is oriented along the beam direction, the ion penetration depth is larger, which, in turn, reduces the sputter rate. The resulting surface roughness may be minimized by removing the material with only few cycles. For standard geometries, both of these optimization problems are already targeted with the high-level approach and the pre-defined raster strategies [17]. However, only the low-level approach enables these strategies for arbitrary shapes in combination with adapted beam paths.

Another typical artifact is a shape distortion via beam-induced modifications, possibly due to heat. This is a severe issue especially for ion beam machining with light ions (cf. section “Ion beam machining with light ions”). The ion beam locally introduces energy that needs time to dissipate before the beam reaches the same position and introduces energy again. In the low-level approach, the raster strategy can be customized such that the local dose per time is minimized (e.g., back-stitch or other custom point- or line-hopping approaches). If provided by the manufacturer, flags for beam blanking may be introduced as well. However, the number of beam blanking operations should be kept as low as possible, as it introduces ‘unused’ patterning time and may cause artifacts due to the limited blanking speed.

**Figure 4:**
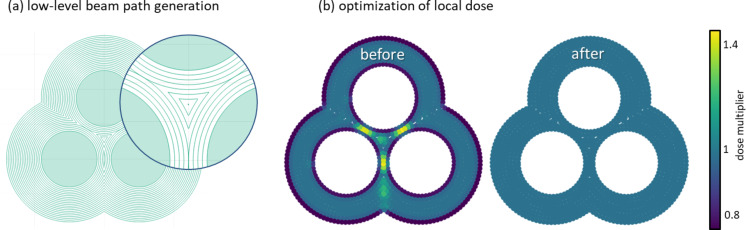
Geometry-optimized patterning. (a) Beam path generation by curve off-setting around arbitrary disconnected shapes. (b) Upon rasterizing curved beam paths, strongly curved regions can exhibit local doses larger than the target dose. This is corrected by a local dose optimization reducing the beam dwell time in these regions.

Once the point cloud of such an adapted beam path is created by rasterization with FIB-o-mat, regions of higher local point densities may appear. This occurs for large curvatures in the off-set curves as schematically depicted in Figure 4a. Here, the actual ion beam profile has an important influence as it defines how much the local dose deviates from the target dose. The local dose received at each spot location equals the sum of the dose applied there and the doses reaching that location from nearby points. By assuming a Gaussian profile of the beam, the local dose can be calculated by integrating over the profiles of all contributing spot locations. In the regions of higher point densities, this results in a local dose exceeding the target dose. For local dose correction, FIB-o-mat first rounds off regions of high curvature in the beam path by replacing them with circle segments. It is recommended to use radii of half the pitch for rounding to keep sufficient information about the geometry. In a second step, the dwell times of individual spot locations are reduced in an iterative manner such that the target dose can be achieved. Figure 4b shows the application of the local dose correction for a trimer geometry. Here, the local dose exceeded the target dose by about 50% before the optimization routine.

### 3 Programming and file formats

The FIB-o-mat toolbox is mainly written in Python and partly in C++. It can be compiled on any modern operating system. The modular layout of FIB-o-mat makes it easily extendable by any FIB user. Currently, only He ion beam patterning is supported, and all manufacturer-specific tools are not publicly available. In the future, it is planned to introduce and share back ends for microscopes of different manufacturers.

As the definitions of geometric primitives and raster styles change between different patterning tools, the corresponding high-level functions need to be implemented by the user if not yet present. All standard primitives and raster styles are available in FIB-o-mat. Alternatively, the low-level approach may be used for full flexibility of the beam path. During import of the graphics, due to the complexity of the .dxf file format, only a subset of features is supported in FIB-o-mat. Similar manufacturer-specific challenges occur during automation. Some manufacturers provide automation tools that can be accessed by the patterning software, others require the use of an API that may even have to be licensed. In the latter case, implementing automation using FIB-o-mat can be highly demanding, for example if the featured APIs are relatively restricted and based on a proprietary programming language.

Another technical challenge concerns the obtained pattern sizes for the low-level approach in FIB-o-mat. Complex patterning tasks easily result in millions of points that may cause memory problems when loaded into the patterning engine of the microscope. Here, a binary file format is highly desirable instead of ascii files with plain text.

## Results and Discussion

Three test cases were selected to demonstrate the different capabilities of FIB-o-mat. These include high-level patterning with automation of magnetic multilayers, low-level patterning with automation of suspended graphene and low-level patterning and beam path optimization for gold on glass. Patterns for all three test cases are available in the git repository of the FIB-o-mat package [18].

### 1 Magnetic patterning of Co/Pt multilayer films

**Used FIB-o-mat features:** high-level beam path generation with automation via stage control.

Magnetic thin films and multilayers are of great technological interest as platforms for spin-based devices encoding bits using nanoscale domain walls or skyrmions [4]. The magnetic properties of thin magnetic films and multilayers can directly be modified in a controlled manner by low-dose ion irradiation. Local variation of the dose using masks or focused ion beams leads to pure magnetic patterning without affecting the topography of the films [32]. The local modification of the magnetic properties, in particular anisotropy and exchange coupling (including the chiral Dzyaloshinskii–Moriya interaction), originates from structural modifications, such as interface structure, atomic ordering, atomic composition, and crystallographic phase [33–34].

Here, the impact of He irradiation on the ferromagnetic multilayer [Co_0.6_/Pt_0.8_]_15_ is studied [35–36]. This multilayer shows perpendicular magnetic anisotropy arising from the Co/Pt interfaces and forms nanometer-scale, labyrinth-like domains with opposite out-of-plane magnetization in remanence. We are particularly interested in how ion irradiation changes the morphology of the magnetic domains and how it influences the nucleation and annihilation of domains in a typical adiabatic field cycle as well as after picosecond laser excitation [37–38].

The fabrication of the samples starts by depositing the magnetic multilayer onto 150 nm thick silicon nitride membranes via magnetron sputtering. The membranes are almost transparent for soft X-rays, which are later employed for imaging of the samples. Subsequently, the multilayer film is locally modified by He irradiation, employing different patterning layouts and varying the applied He ion dose. Typical patterns comprise checkerboard structures, lines, and dots with characteristic dimensions ranging from 50 nm to several micrometers. The applied He dose varies between 10 and 50 ions/nm^2^. The the appropriate dose range was determined in prior automated dose tests (cf. Supporting Information File 1, section “Automation in FIB-o-mat”). The used ion beam current was 2.6 pA at an acceleration voltage of 30 kV and an extractor voltage of 32 kV. The dwell time was 1 μs and the pitch was 5 nm.

The magnetic nanometer-scale domain configurations were imaged via soft X-ray holography [39], exploiting X-ray magnetic circular dichroism [40] and giving rise to an absorption contrast of areas with different out-of-plane magnetization.

**Figure 5:**
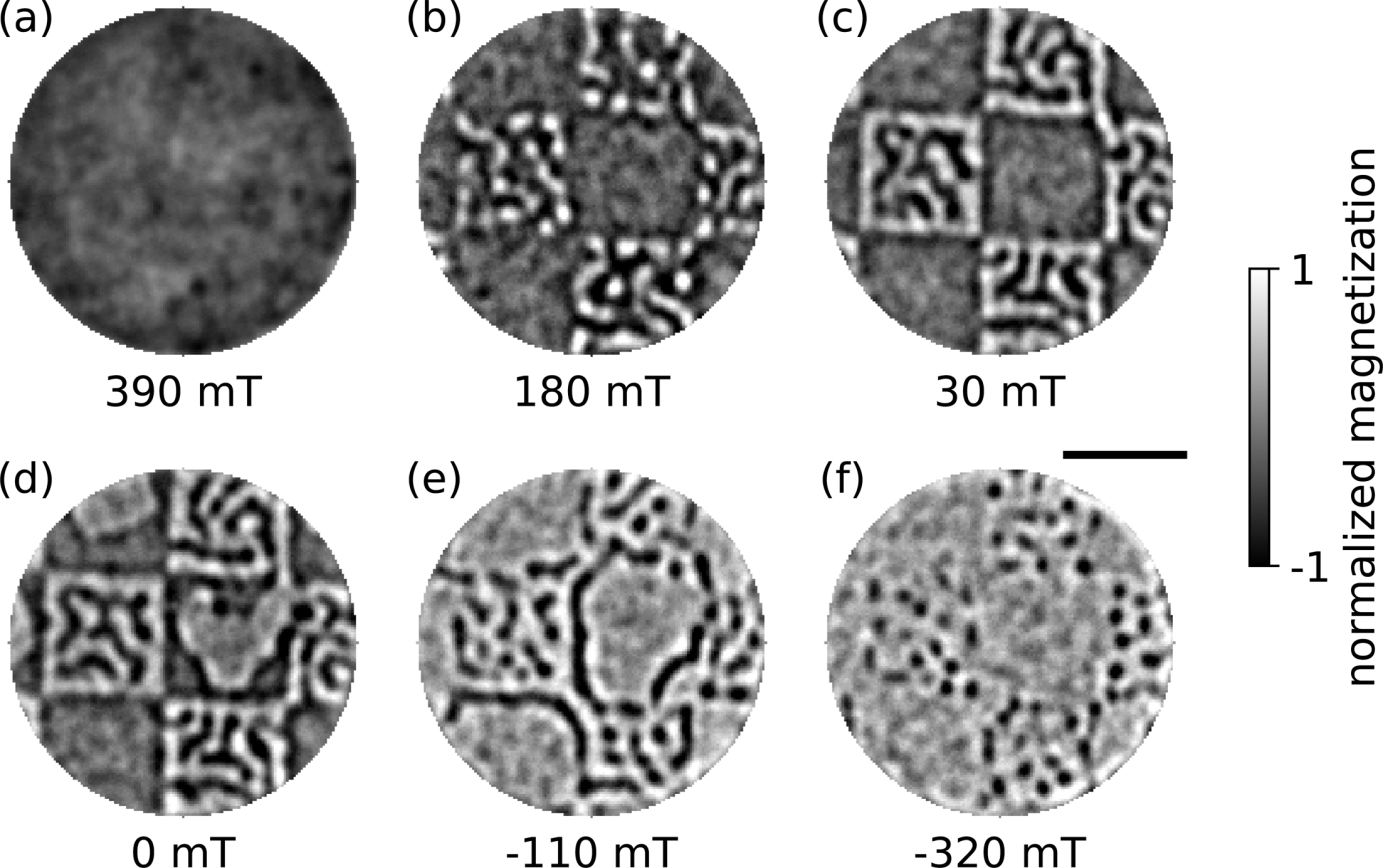
Magnetic nanopatterning of a Co/Pt multilayer. Fourier-transform holography (FTH) images (a–f) with a predefined field of view (FOV) of 1.5 μm illustrate a magnetic field cycle from positive saturation at 390 mT to negative saturation in an He ion irradiated sample, revealing a checkerboard pattern with 500 nm side length. The black–white contrast encodes domains with opposite out-of-plane magnetization. Starting from a saturated state (a), magnetic domains first form in ion-irradiated regions (b, c) before they appear in non-irradiated regions (d). The non-irradiated regions saturate earlier when the applied magnetic field is further decreased (e, f). The presented magnetic film, thus, supports the coexistence of regions with different magnetic properties. The scale bar is 500 nm.

Areas irradiated with an ion dose of 50 ions/nm^2^ no longer show an out-of-plane magnetization component. Based on previous experiments, we assume that the interfaces inside the multilayer are modified so severely that the magnetization switched to in-plane [32–35]. In contrast, an ion dose of 10 ions/nm^2^ does not lead to a modification of the multilayer that is observable in the domain pattern or in the hysteresis of the sample. Similar ion dose ranges are reported elsewhere for Co/Pt multilayer systems on SiO_2_ substrates [41]. Figure 5 shows images of the domain configuration in the multilayer irradiated with an ion dose of 30 ions/nm^2^. The gray-scale images encode purely magnetic contrast with white and black colors indicating opposite out-of-plane magnetization. The sample was irradiated in a checkerboard-like pattern with a side length of each square of 500 nm and a total area irradiated of 20 × 20 μm. In this sample, the domain size in irradiated regions is significantly reduced as compared to domains in non-irradiated regions, as shown in Figure 5d (at 0 mT). Tailored modification of the magnetic film thus enables the coexistence of two magnetic structures in a checkerboard pattern, revealing different magnetic behavior side by side. The sample was imaged in a full applied-field sweep from saturation at positive field to saturation at negative field. During this measurement, images at particular applied fields were recorded (Figure 5). After saturating the magnetic film (at 390 mT, Figure 5a) and reducing the applied field, domains form (180 mT, Figure 5b) and grow (Figure 5c) first in irradiated regions, before domain formation in the non-irradiated regions starts from the edges of the checkerboard squares (0 mT, Figure 5d). Analogously, the non-irradiated regions saturate first at (−110 mT, Figure 5e), and the irradiated regions saturate later at less than (−320 mT, Figure 5f). Remarkably, the domains in the irradiated areas shrink to a dense array of small bubble domains close to the saturation point (Figure 5f). Hence, through He-assisted sample fabrication, the formation of magnetic domains can be enhanced in a controlled manner, probably due to the increased density of pinning sites, that is, variations of the local anisotropy.

Since the influence of different ion doses and pattern shapes on the formation of magnetic domains is not known a priori for different magnetic material systems, a lot of different combinations of doses and shapes must be patterned initially (cf. Figure S2, Supporting Information File 1, where in total 42 different patterning sites are shown of which each site includes of a different shape/dose combination). Using the FIB-o-mat package, the creation of the patterns can be automated. The patterning is carried out with the automation tool of the NPVE software controlling the He ion microscope according to the FIB-o-mat patterns. No further manipulation of the patterns by the user is required. Hence, FIB-o-mat is technically not necessary for this task but constitutes a significant time-saver.

### 2 Mechanical resonators based on suspended single-layer graphene

**Used FIB-o-mat features:** low-level beam path generation and automation via stage control, patterns optimized for speed to generate a large number of trampolines with varying bridge widths.

Atomically thin graphene has extraordinary mechanical properties [3], and graphene nanomechanical resonators have been employed as various sensors [42–46]. Yet, the sensitivity at room temperature is limited by a rather low quality factor. Patterning of the devices into trampoline-shaped resonators yields a large increase in quality factor and, thus, device performance [47–48]. Furthermore, the material removal reduces the thermal coupling of the central device area to the supporting substrate. As graphene is a single-atomic layer, it exhibits the lowest possible heat capacity per unit area of the material [48]. Its broadband spectral absorbance [49], in combination with a thermal stability up to 2600 K [50], renders graphene an exciting candidate for room-temperature bolometry [51].

Single-layer graphene was grown by chemical vapor deposition onto a multicrystalline copper foil using methane as precursor gas at 1035 °C. For the transfer process, the graphene sheet was covered by a 500 nm thick PMMA layer. After etching the copper foil, the graphene sheet was transferred onto a SiN membrane with a regular grid of holes. The transfer process is described in detail elsewhere [52]. The SiN membrane was covered with a thin layer of gold, which allowed us to electrically contact the graphene sheet and actuate the resonators electrostatically. The motion of the devices is detected using a Michelson interferometer [48].

**Figure 6:**
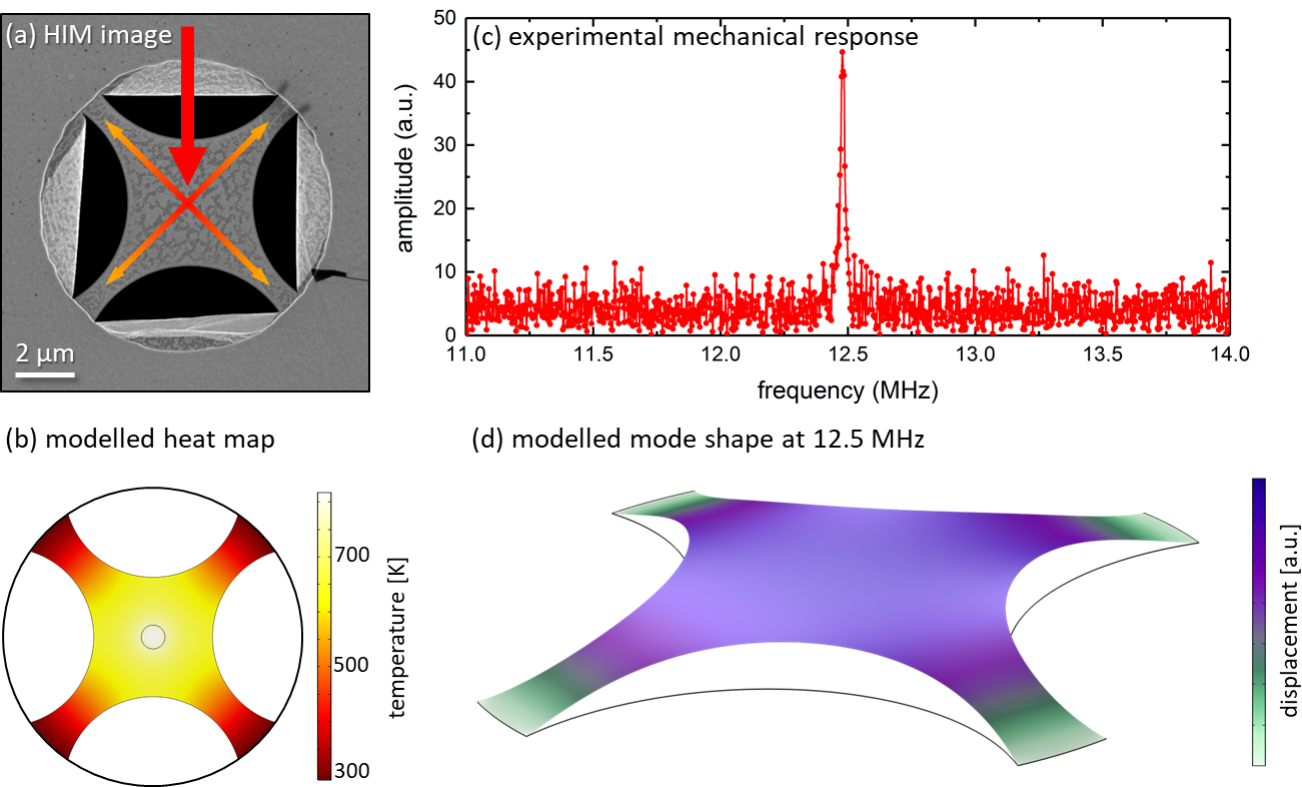
Single-layer graphene resonator. (a) Secondary electron He ion microscopy image of the graphene resonator patterned into a trampoline geometry. Laser excitation from the top is depicted schematically by the red arrow. (b) The corresponding heat distribution induced inside the trampoline due to the laser heating was obtained by finite element modelling. The resonator is excited by electrical gating and the displacement is detected in an interferometric read-out. (c) Experimentally determined frequency response of the fundamental mode of the trampoline resonator at 12.5 MHz with (d) the simulated mode shape of the fundamental resonance.

Figure 6a depicts a secondary electron HIM image of a patterned trampoline graphene resonator. A He ion current of 3 pA was employed at an acceleration voltage of 30 kV and a BIV of around 32.3 kV. The patterns were constructed from circle segments combined with linear segments to define length and width of the trampoline bridges. In the absence of contaminants, a pitch of 50 pm in combination with a dwell time of 4 ms leads to well-defined single-repeat cuts. To avoid ion beam-induced modifications of the graphene sheet, the pattern location was adjusted at one of the SiN holes followed by automated stage moves and patterning. Thus, a large number of trampoline resonators could be fabricated without unintended beam impact due to imaging in a reasonable overall patterning time. The corresponding step-and-repeat list with the geometric primitives, path settings, and displacements was created in FIB-o-mat and could be directly loaded into the patterning engine of the microscope.

To probe the device, a laser spot with a width of around 1.3 μm at a wavelength of 632 nm was directed onto the central region of the resonator. A numerical description of the locally heated resonator was carried out by finite element modeling. The obtained heat map shows good thermal decoupling of the trampoline geometry with a large gradient. The central region exhibits temperatures around 800 K while the bridges stay close to room temperature. The resonance frequency of the fundamental mode is 12.5 MHz (cf. Figure 6b) and exhibits a quality factor of about 300 without laser heating. When suspended graphene is heated by a light source, the built-in tension in the device is reduced because graphene has a negative thermal expansion coefficient [53], and a down-shift in resonance frequency is observed. The mode frequency can be shifted up by 100 percent through increasing the laser power from 15 to 150 μW. The large bandwidth in combination with the increased responsiveness created by the trampoline pattern allows the system to operate as an ultra-sensitive and ultra-fast bolometer [48]. Finally, the increased quality factor and reduced thermal coupling to the substrate should allow for efficient side-band cooling experiments, which so far are hindered by very strong thermal coupling and low quality factors [54–55].

### 3 Plasmonic tetramer antennas based on single-crystalline gold flakes

**Used FIB-o-mat features:** low-level beam path generation with optimization concerning heat transport, patterning time, and local dose.

**Figure 7:**
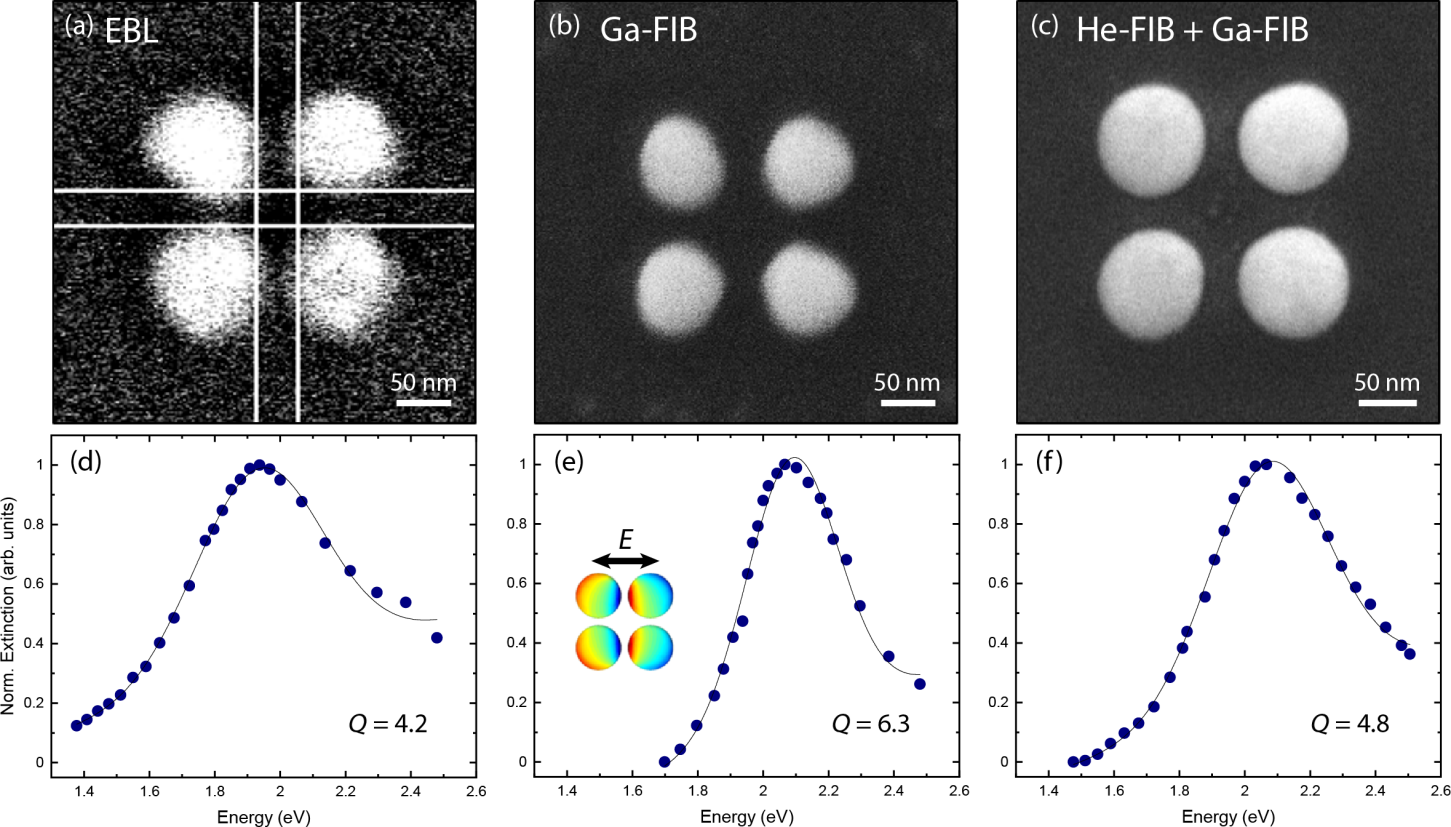
Coupled plasmonic antennas. (a–c) Scanning electron micrographs of gold tetramers on glass with a target geometry of 45 nm particle radius and 35 nm gap width, fabricated by different techniques: (a) by electron beam lithography, (b) Ga ion beam machining, and (c) He ion beam machining of the tetramer geometry and removal of surrounding gold by Ga FIB. The low image resolution in (a) is caused by the difficulties of imaging isolated tetramers on a non-conductive glass substrate. (d–f) Normalized extinction (sum of scattering and absorption) of the tetramers. The inset depicts the charge distribution of the excited plasmonic mode upon incidence of linearly polarized light.

Thin flakes of single-crystalline gold constitute an ideal platform for plasmonic applications due to the lack of scattering losses at grain boundaries and surface roughness [56]. In nanostructured gold, collective excitations of the free electron gas may occur under the incidence of visible light [2]. These plasmon polaritons of individual nanoparticles are not only strongly resonant with the ability to concentrate light below the diffraction limit. They may also be efficiently coupled in closely packed ensembles of plasmonic particles. In the case of three-dimensional crystals from gold spheres even deep strong coupling of light and the plasmonic excitation at room temperature was achieved [57]. Here, we investigate the influence of the fabrication routine on the resonant response of small ensembles of coupled plasmonic scatterers, namely tetramers of spherical gold discs residing on a glass substrate. Such ensembles may feature dipole-forbidden eigenmodes [58] with low damping when being excited by the incidence of structured light [59]. First, we focus on comparable tetramer geometries to assess the influence on geometric fidelity and possible ion-beam induced material/substrate modifications on the plasmonic response by measuring the bright optical modes [59]. The target geometry for all fabrication techniques is a particle radius of 45 nm with a gap size of 35 nm. This is the geometry that can reliably fabricated by resist-based electron beam lithography on physically sputtered gold layers (cf. Figure 7a). Later, we will assess the ultimate resolution of He ion beam machining to minimize the gap sizes.

The gold flakes of approx. 30 nm thickness were wet-chemically synthesized on glass [60] and transferred to the target substrate via a PMMA-mediated method. On the target glass substrate, a thin layer of gold was sputtered with two empty square windows of 100 × 100 μm^2^ in size, where the flakes were placed. The fabrication of the tetramer structures was carried out using Ga ion beam milling only, or a combination of He ion beam milling for the definition of the tetramer and Ga ion beam milling for the large-area removal of the surrounding gold. Figure 7b,c depicts the corresponding scanning electron beam micrographs. The limited image quality of the lithographically defined tetramer is caused by the fact that the isolated structures are located on a glass substrate, leading to severe charging effects. For Ga ion beam milling a current of 10 pA, a pitch of 3 nm and a dwell time of 1 μs were employed in a two-step patterning process. First, the surrounding gold was removed by rectangular scanning of a square of several micrometers from which a slightly larger tetramer shape was subtracted. Subsequently, the remaining tetramer was polished with ring shapes. During He ion beam patterning, first, the tetramer was patterned with a large outline of 40 nm thickness using an optimized low-level beam path defined in FIB-o-mat (cf. Supporting Information File 1, section “Challenges in the patterning of the plasmonic tetramers”). The beam path had a curve off-set of 0.25 nm. The pitch was 0.25 nm around the tetramer and 0.5 nm inside the tetramer. The dwell time was 5 μs, and the pattern was repeated 12 times. The employed beam current was around 2 pA at an acceleration voltage of 30 kV and a BIV of 32 kV. The second step was the removal of the surrounding gold with Ga using again a shape obtained by a Boolean operation.

Figure 7 shows that shape distortions mainly occur for the lithographically defined and the Ga FIB-fabricated tetramers. However, also in the case of the He ion beam-patterned tetramers the subsequent removal of gold using the Ga ion beam leads to slight distortions that were not present before. This is caused by the typical tails in the beam profile of a focused Ga ion beam [19]. Optical investigation was carried out using visible-light spectroscopy in a transmission spatial modulation setup. The spatial displacement of the focus modulates the signal [61] and the lock-in coupled detector provides an excellent signal-to-noise ratio for individual small scatterers.

Figure 7d–f shows the extinction (sum of scattering and absorption) spectra corresponding to the tetramers in Figure 7a–c, respectively. The extinction was measured for plane wave incident light of linear polarization. The polarization was chosen along the *x* axis as depicted in Figure 7e. This configuration and any other in-plane polarization excite the optically active *E_u_* mode of the tetramer [59]. The plasmonic resonance is significantly broadened for the lithographically defined tetramer. Interestingly, the highest *Q* factor (*Q* = ω_res_/δω with the resonance frequency *Q* = ω_res_ and the resonance width δω) was achieved for the tetramers obtained by pure Ga patterning, even though the monomer shape is not perfectly round. Each monomer acts as a small dipole in our measurement configuration and its response is not very sensitive to its actual shape as long as surface roughness does not increase the scattering losses. Finite-difference time-domain modeling taking into account the slightly varying geometries led to numerical Q factors of *Q*_num_ = 3.3 for tetramers fabricated by EBL, *Q*_num_ = 5.4 for Ga FIB, and *Q*_num_ = 4.8 for He FIB. This is in reasonable agreement with the experimental results.

**Figure 8:**
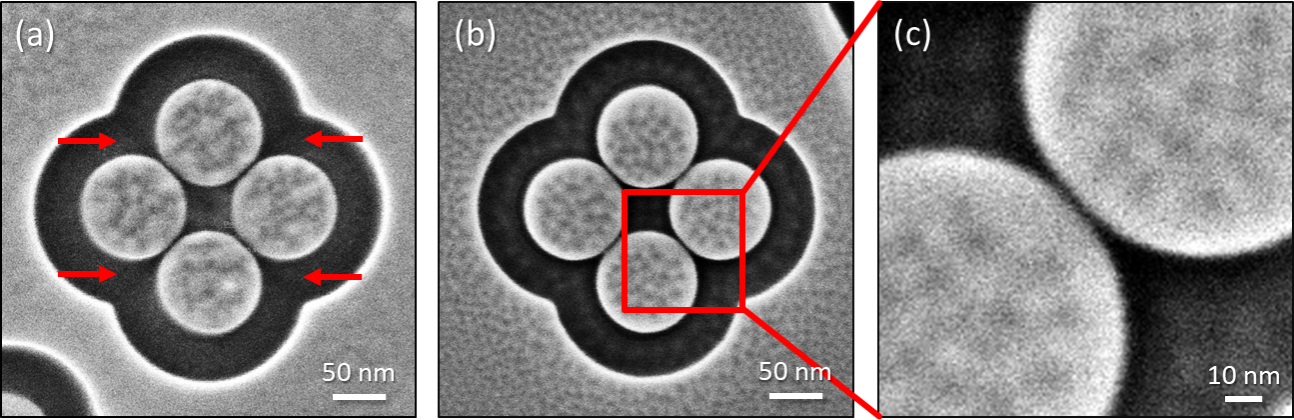
Optimized tetramer. Secondary electron He ion microscopy images of gold tetramers on glass with minimized gaps after certain steps of optimization. (a) The gap size was defined by a single line resulting in gap sizes of only 3 nm. The high-curvature regions cause an increased milling depth marked by red arrows. (b) After local dose correction, these trenches disappeared. (c) The close-up of (b) proves the remarkable high pattern fidelity with a gap size of about 3 nm.

The mutual coupling of the individual monomers, however, strongly depends on the interparticle distance due to the evanescent decay of the plasmonic near field. Hence, a nanopatterning approach that is able to realize sub-10 nm gaps in a reproducible manner is highly desired. He ion beam milling already demonstrated these capabilities in the fabrication of strongly coupled dimers with gap distances of less than 6 nm [62–64]. In all previous cases, the antenna shapes were pre-fabricated with Ga ion beam milling or lithographic approaches and only single-line cuts were performed with He ions to create dimer antennas. Here, we prove the ultimate resolution capabilities for the tetramer geometry with minimized gap sizes patterned in a gold flake of about 40 nm thickness using He beam milling for the complete antenna and not only for separating individual parts of a pre-fabricated antenna. Figure 8a depicts the secondary electron HIM image of a tetramer patterned using a similar low-level beam path as used for the tetramer in Figure 7c. As in the example above, the beam path was optimized regarding reduced heat damage and redeposition. However, the gap distance was defined by a single path between the monomers. Most importantly, this resulted reliably in gap sizes between 3 and 4 nm when the gold surface was free of contaminants and when optimum ion beam conditions were achieved. The mechanical He column adjustment was optimized such that almost no electronic beam tilt or shift corrections were necessary and a high spot control of 6 could be used for the 20 μm aperture at a standard gas flux of 2e^−6^ Torr. The thereby obtained beam current reached values between 1.2 and 1.8 pA, depending on the actually employed trimer.

However, this specific geometry results in large curvatures of the path when approaching the gaps. The red arrows in Figure 8a indicate regions of larger depth arising due to the locally increased dose. Local dose correction was performed as described in subsection 2 of “The FIB-o-mat toolbox” and enabled uniform milling down to the glass substrate. It has to be mentioned that the actual results of the patterning of thin gold flakes on glass varied strongly, depending on the cleanliness of substrate and gold flake (cf. Supporting Information File 1, section “Challenges in the patterning of the plasmonic tetramers”). Nevertheless, we show here, for the first time, high-fidelity patterning of plasmonic nanostructures with geometrical details as small as 3 nm. This is mainly achieved by defining a contour-parallel and dwell time-optimized beam path using FIB-o-mat. To the best of our knowledge, such geometry-adapted beam paths cannot be created by current patterning software provided by the instrument manufacturers. Furthermore, FIB-o-mat enables the rapid generation of different patterns with varying path geometries, local dwell times and pitches, as well as number of repetitions allowing for efficient patterning iteration and optimization.

## Conclusions and Outlook

He ion beam machining offers exciting prospects for the fabrication of devices from two-dimensional and quasi two-dimensional materials. The extremely small spot size, large depth of field, and high stability render the He ion beam ideal for automated high-fidelity patterning in cases where low sputter rates are negligible. This holds especially true for two-dimensional materials where only small amounts of material have to be removed while guaranteeing highest patterning precision. Since He ion microscopy is a relatively new technique, its full potential still has to be exploited. Here, we contributed in developing the advanced pattern creation toolbox FIB-o-mat [18]. FIB-o-mat has a Python interface and a fully documented modular structure, such that it is easy to use and easy to extend. In its current version, it provides high-level pattern creation, low-level beam path generation, as well as optimization and automation tools for He ion beam machining. The aim is to extend FIB-o-mat such that the tools can be used with microscopes of any manufacturer, covering ion beam machining with a variety of ions.

For the efficient use of FIB-o-mat, the following workflow may be employed: (i) Define the geometry to be patterned. (ii) Define the beam path such that it follows the edges of the geometry, for example, by using the curve off-setting tool where required. (iii) Define a rasterization strategy in a way that artifacts, such as redeposition and other beam-induced effects, are minimized. (iv) When long patterning durations are necessary, vary the raster parameters (and/or increase the beam current). (v) In the case of curvatures in the beam path, use the local dose optimization to achieve a uniform target depth.

To demonstrate the capabilities of FIB-o-mat, three different 2D material systems were patterned. Multilayers of Co/Pt were modified regarding their local magnetic response without changing their topography. Suspended single-layer graphene was cut into trampoline-shaped mechanical resonators, and single-crystalline gold was patterned into coupled plasmonic antennas. In the former two cases, the ease to vary the geometry and patterning parameters in an automated way greatly facilitated both the systematic patterning optimization and a detailed analysis of the device properties. While these two cases pattern creation and automation can, in principle, be carried out using the software of the microscope manufacturer, it is impossible to create the adapted beam path for the tetramer geometry by any commercial patterning software. The realized minimum gap size of 3 nm for a gold thickness around 40 nm and the perfect spherical shapes of the monomer discs are not obtainable without beam path optimization, nor can they be obtained by any other fabrication technique. Hence, the low-level approach in FIB-o-mat provides the necessary functionalities to unlock the ultimate performance of He ion microscopy.

## Supporting Information

File 1 Additional experimental data.
