# Supplementary material for: The patterning toolbox FIB-o-mat: Exploiting the full potential of focused helium ions for nanofabrication
Source: Beilstein J Nanotechnol. 2021 Apr 6;12:304–18. doi: 10.3762/bjnano.12.25 (PMC8042487; doi:10.3762/bjnano.12.25)
Supplement: File 1 — Additional experimental data. [file Beilstein_J_Nanotechnol-12-304-s001.pdf]

BEILSTEIN JOURNAL OF NANOTECHNOLOGY

Supporting Information

for

The patterning toolbox FIB-o-mat: Exploiting the full potential of focused helium ions for nanofabrication

Victor Deinhart, Lisa-Marie Kern, Jan N. Kirchhof, Sabrina Juergensen, Joris Sturm, Enno Krauss, Thorsten Feichtner, Sviatoslav Kovalchuk, Michael Schneider, Dieter Engel, Bastian Pfau, Bert Hecht, Kirill I. Bolotin, Stephanie Reich and Katja Höflich

Beilstein J. Nanotechnol. **2021,** *12,* 304–318. doi:10.3762/bjnano.12.25

Additional experimental data

License and Terms: This is a supporting information file under the terms of the Creative Commons Attribution License (https://creativecommons.org/licenses/by/4.0). Please note that the reuse, redistribution and reproduction in particular requires that the author(s) and source are credited and that individual graphics may be subject to special legal provisions.
The license is subject to the *Beilstein Journal of Nanotechnology* terms and conditions: (https://www.beilstein-journals.org/bjnano/terms)

FIB-o-mat overview

In the following, a brief overview of the FIB-o-mat Python package is given. For a more detailed overview, we refer to the documentation and git repository [1,2]. Figure S1 visualizes the overall class structure of the package. This tree-like structure follows closely the usual workflow existing in GUI-based patterning software. The root of the package is the ‘Sample’ class. All other objects are added to this class directly or indirectly. ‘Sites’ can be added to a ‘Sample’ and represent a fixed stage position and field of view of the microscope. Each ‘Site’ can hold a arbitrary number of ‘Patterns’, which will be patterned at the corresponding ‘Site’. ‘Patterns’ are composed of a ‘Shape’, defining the geometry, a ‘Mill’ class, holding information about the ion beam (dwell time, current, and optionally a beam profile), and a ‘RasterizationStyle’, which specifies how the ‘Shape’ will be rasterized. Patterns not only hold single ‘Shapes’ but also groups of shapes (‘Groups’), which share the patterning settings. Additionally, ‘Shapes’ and ‘Patterns’ can be arranged in regular grids, which are created by the ‘GridGenerator’ class. Within this class, properties of a pattern can be changed depending on the position of a ‘Pattern’ in the grid. For example, the dwell time could be changed in the horizontal direction while the pitch is varied in the vertical direction.

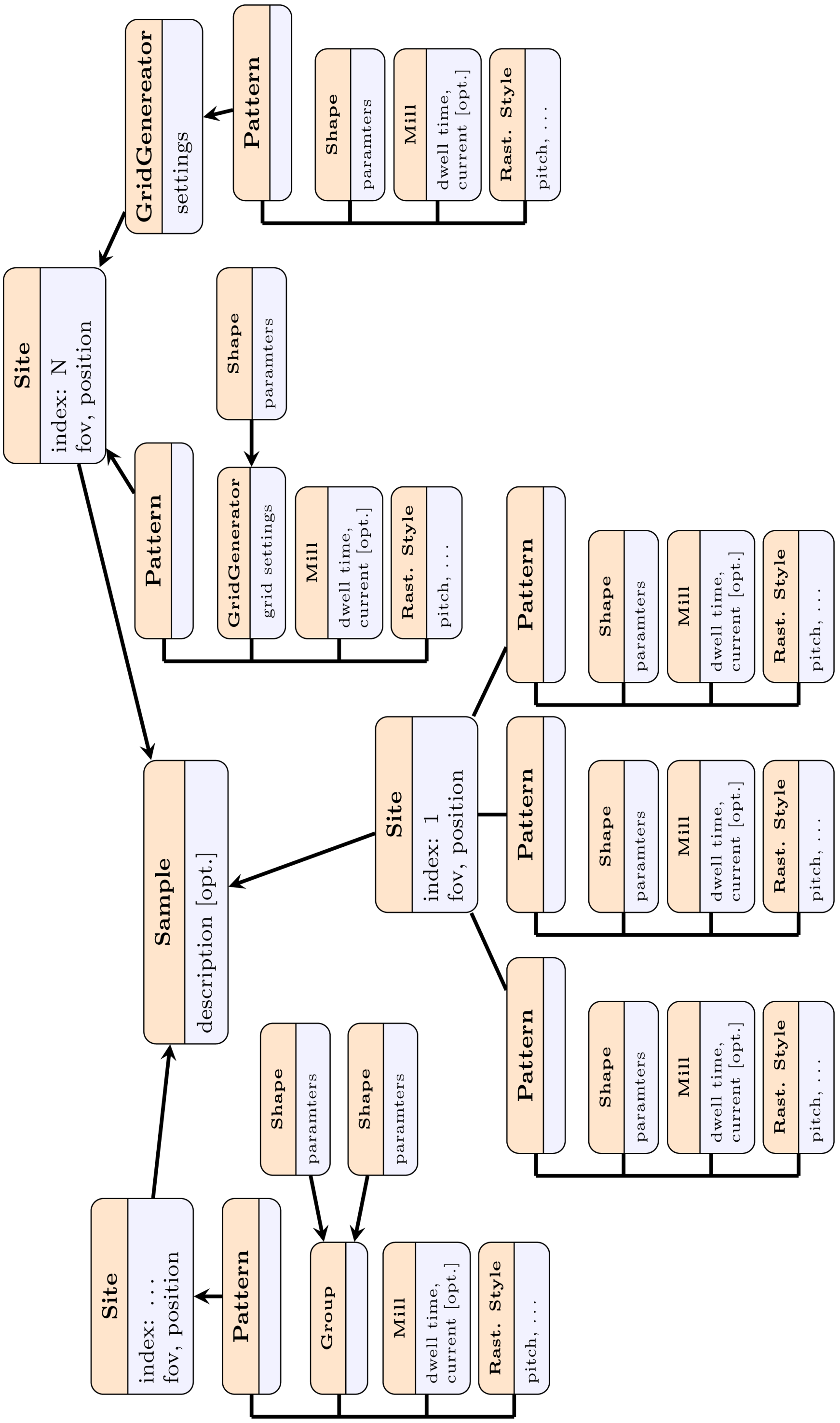

Figure S1: Overview of the class structure used in the FIB-o-mat Python package.

The code in Listing 1 below gives a simple usage example. A single patterning site containing a line is created.

```python
from fibomat import Sample, Mill, Q_, U_
from fibomat.shapes import Line
from fibomat import raster_styles
from fibomat.default_backends import SpotListBackend

# create a Sample class object with optional description
sample = Sample(description='Useful description here')

# add a site to the sample with center = (0, 0) and field of view of (10, 10)
site = sample.create_site(dim_position=[0, 0] * U_('µm'), dim_fov=[10, 10] * U_('µm'))

# create a Pattern with a Line shape and add it to the site
site.create_pattern(
    dim_shape=Line((-5, 0), (5, 0)) * U_('µm'),
    mill=Mill(dwell_time=Q_('5 ms'), repeats=1),
    raster_style=raster_styles.one_d.Curve(pitch=Q_('1 nm'), scan_sequence=raster_styles.ScanSequence.CONSECUTIVE)
)

# export a rasterized version of the pattern as text file in a pre-defined (but editable) format. See docs for details.
sample.export(SpotListBackend).save('pattern.txt')

# plot the pattern
sample.plot()
```

Listing 1: Code listing shows how a simple pattern is created with the FIB-o-mat package.

Automation in FIB-o-mat

Figure S2 shows a plot of the patterns used in the first test case in section "Magnetic patterning of Co/Pt multilayer films" of the main manuscript, where membranes of 250 μm $\times$ 250 μm were patterned. For this, the built-in automation tool of the NPVE patterning software was used. Each blue rectangle in Figure S2 denotes a single patterning site. With the help of the automation tool in NPVE, stage movements and adjustments of the field of view could be made independently of the user (called 'step and repeat list' in NPVE). The input file for this was generated within FIB-o-mat with the help of a customized export back end. Note, that this back end is currently not included in the open source code. It will be added to the open source code in the future once all difficulties concerning (software) licenses are fully resolved.

In general, it should be possible to automate the patterning process for other patterning systems besides NPVE/Zeiss Orion Nanofab. The only requirement is that the software offers some kind of interface that FIB-o-mat can access. For example, Zeiss SmartSEM and SmartFIB offer a Python-callable API. FIB-o-mat can be used to generate patterns on the fly, which, in turn, can be directly loaded into the patterning system via the API.

Figure S2a shows a plot example, which is an interactive HTML document. Yellow shapes indicate the patterning geometry. The big blue rectangles show the corresponding positions and fields of view of the patterning sites. It is possible to zoom, pan, and measure distances and angles. Further, on hovering over patterns, information about shape, beam, and rasterizing settings are shown (cf. Figure S2b). The generated HTML document is self-supported and can be transferred and viewed independently of the FIB-o-mat package.

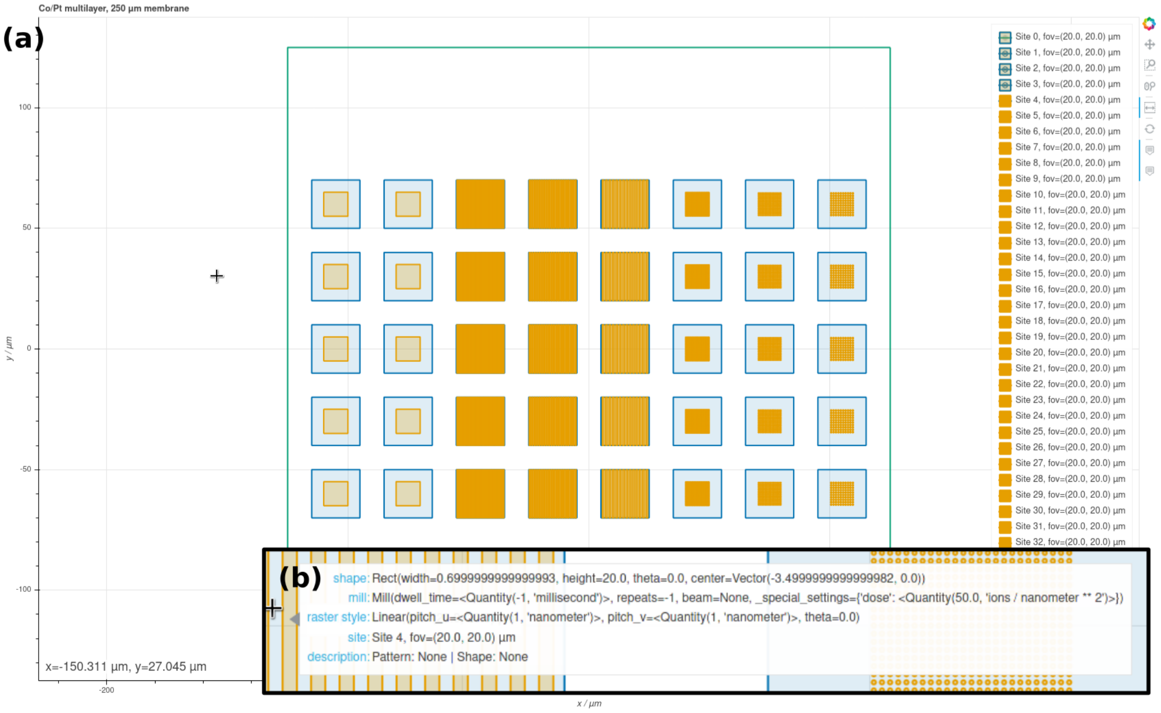

Figure S2: Screenshot of a plot generated within FIB-o-mat used in the first test case Magnetic Patterning of Co/Pt Multilayer Films introduced in section "Magnetic patterning of Co/Pt multilayer films" of the main manuscript.

Challenges in the patterning of the plasmonic tetramers

Depending on the material of a sample and the patterning layout, it may be required to fine-tune the beam paths and the dwell times of individual spots. The exact optimization steps are unique for every sample. Here, some more details on the used optimization methods for the plasmonic tetramer antennas presented in section "Plasmonic tetramer antennas based on single-crystalline gold flakes" of the main manuscript are given.

This application case was especially demanding since, in contrast to suspended 2D films, the full interaction volume in the glass substrate, which is a bad heat conductor, acted as possible heat source and may have triggered further unwanted beam-induced effects. Furthermore, the glass substrate is non-conductive posing a challenge concerning charging artifacts. For the fabrication of the single-crystalline plasmonic tetramers, the Au flake was placed in a free window of an otherwise gold-coated glass substrate such that the overlap with the frame of the window acted as electric contact.

Thus, we avoid charging effects in SEM but also in HIM because the flood gun is not available

for charge compensation during patterning (cf. FigureS3a for patterning an Au flake on bare glass that is electrically not grounded). Intuitively, one would first perform the coarse patterning by Ga FIB followed by edge definition and polishing with He FIB. However, this causes several problems. First, adjusting the corresponding HIM patterns would require a snapshot for proper alignment. This would imply unwanted ion beam impact on the monomers that may modify the material response of the gold structure. The second issue is the mentioned problem regarding the flood gun. When the gold structure is already electrically separated on the glass substrate, a strong drift occurs, making patterning impossible. One can cheat the system by starting the regular scanning and immediately afterwards the patterning engine such that the flood gun "forgets to stop". However, this procedure is very impractical and also introduces a beam impact on the plasmonic structures. Therefore, we decided to first define the tetramers with a broad rim by He FIB placing the patterns blindly on the Au flake. This was followed by the removal of the surrounding gold by Ga FIB.

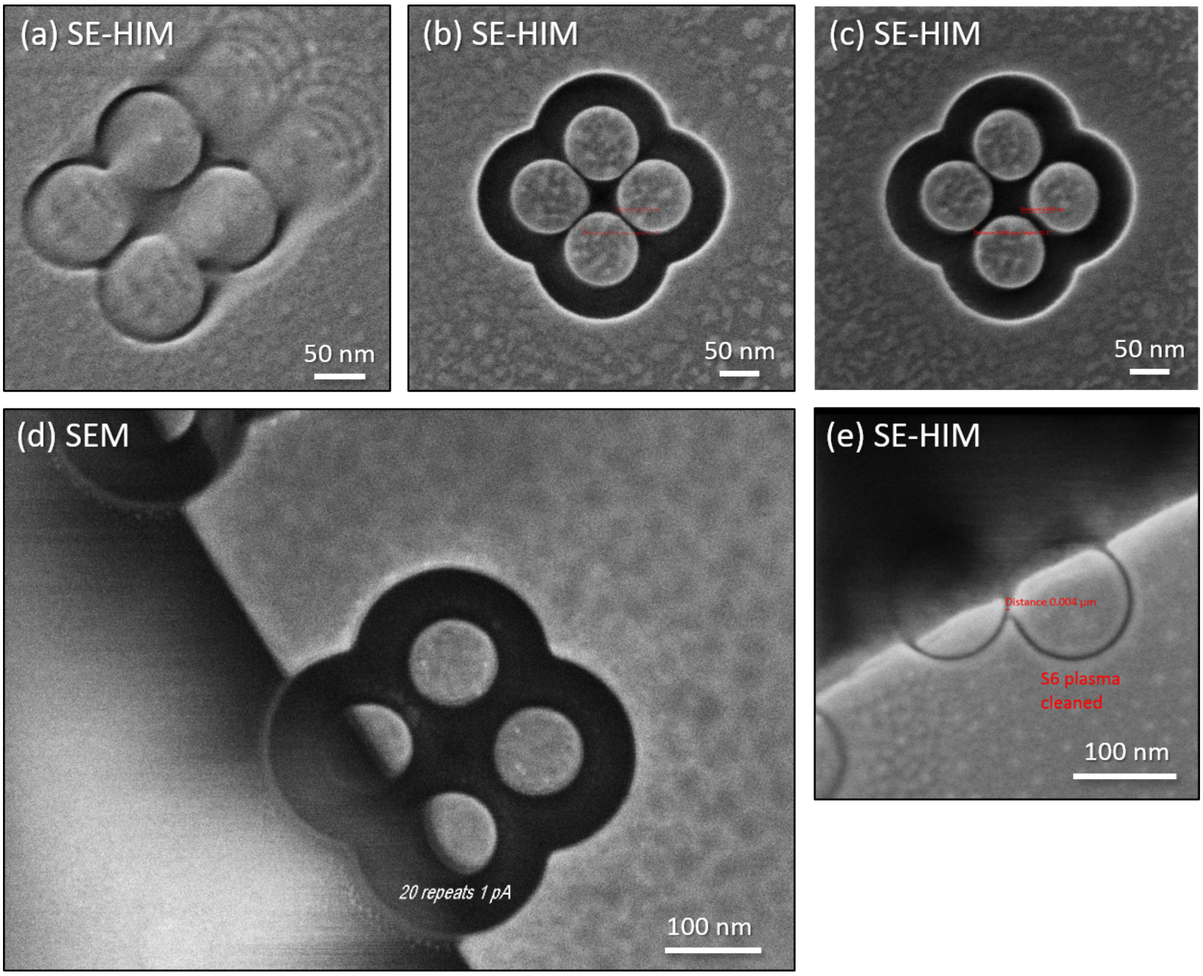

Figure S3: Some of the observed artifacts upon tetramer patterning. (a) Strong drift due to charging of an Au flake that is placed onto a bare glass substrate and is, therefore, electrically not grounded, (b) agglomeration of material in the tetramer center, (c) displacement of separate monomers in the tetramer structure, and (d) shift/moving of material in non-patterned regions. The image was taken via SEM at 15 keV to ensure sufficient material contrast of gold. (e) No shift is observed for a smaller local dose.

The actual He FIB patterning of the gold flakes was equally demanding. As gold exhibits a significant decrease of the melting temperature with decreasing structure size [3], accompanied by a large mobility leading to Ostwald ripening and coalescence of small features [4], heat-induced deformations occur much earlier than in most other materials. In addition, contaminants from the wet-chemical fabrication of the gold flakes, partly situated at the gold/glass interface and, therefore, not removable by cleaning approaches, worsened the patterning situation. Hence, many iterative steps were required to finally realize high-fidelity tetramers.

Used optimizations/settings:

- sample cleaning by oxygen plasma
- many repeats instead of few ones → dwell times not too large
- in regions close to observed beam-induced effects: pitch locally increased
- local dose optimization in regions of high beam path curvature.

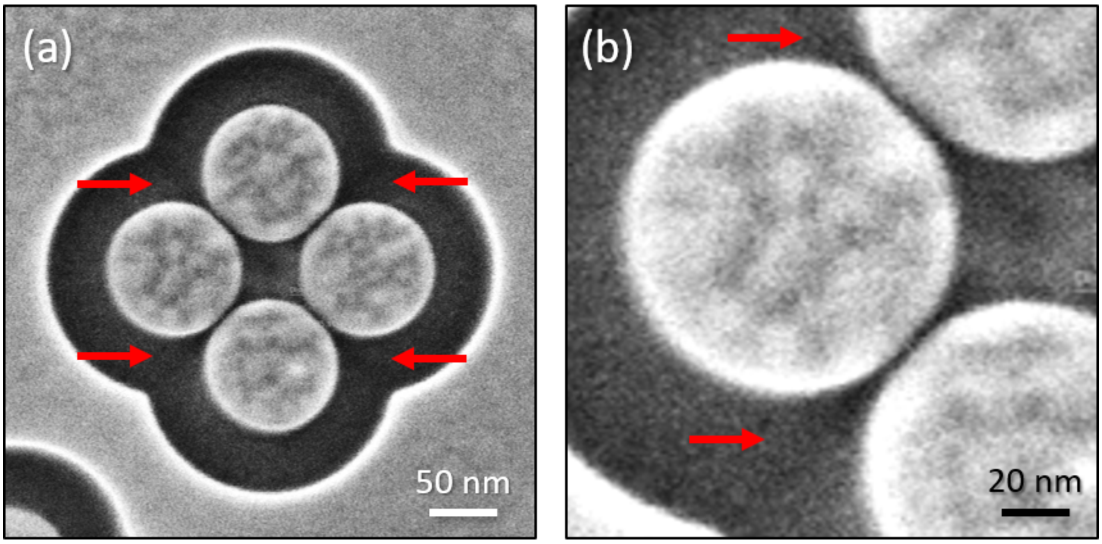

Figure S4: Secondary electron He ion microscopy images of a gold tetramer on glass with minimized gaps. (a) The gap size was defined by a single line resulting in gap sizes of only 3 nm. The high-curvature regions cause an increased milling depth marked by red arrows. (b) Enlarged view of (a) to increase visibility of the local dose effect.

Figure S4 shows an enlarged view of Figure 8 of the main manuscript to increase the visibility of the deeper milling occurring due to higher local doses without local dose optimization. The dose optimization process balances out the local ion fluxes by adjusting dwell times. The corresponding algorithm is based on [5].

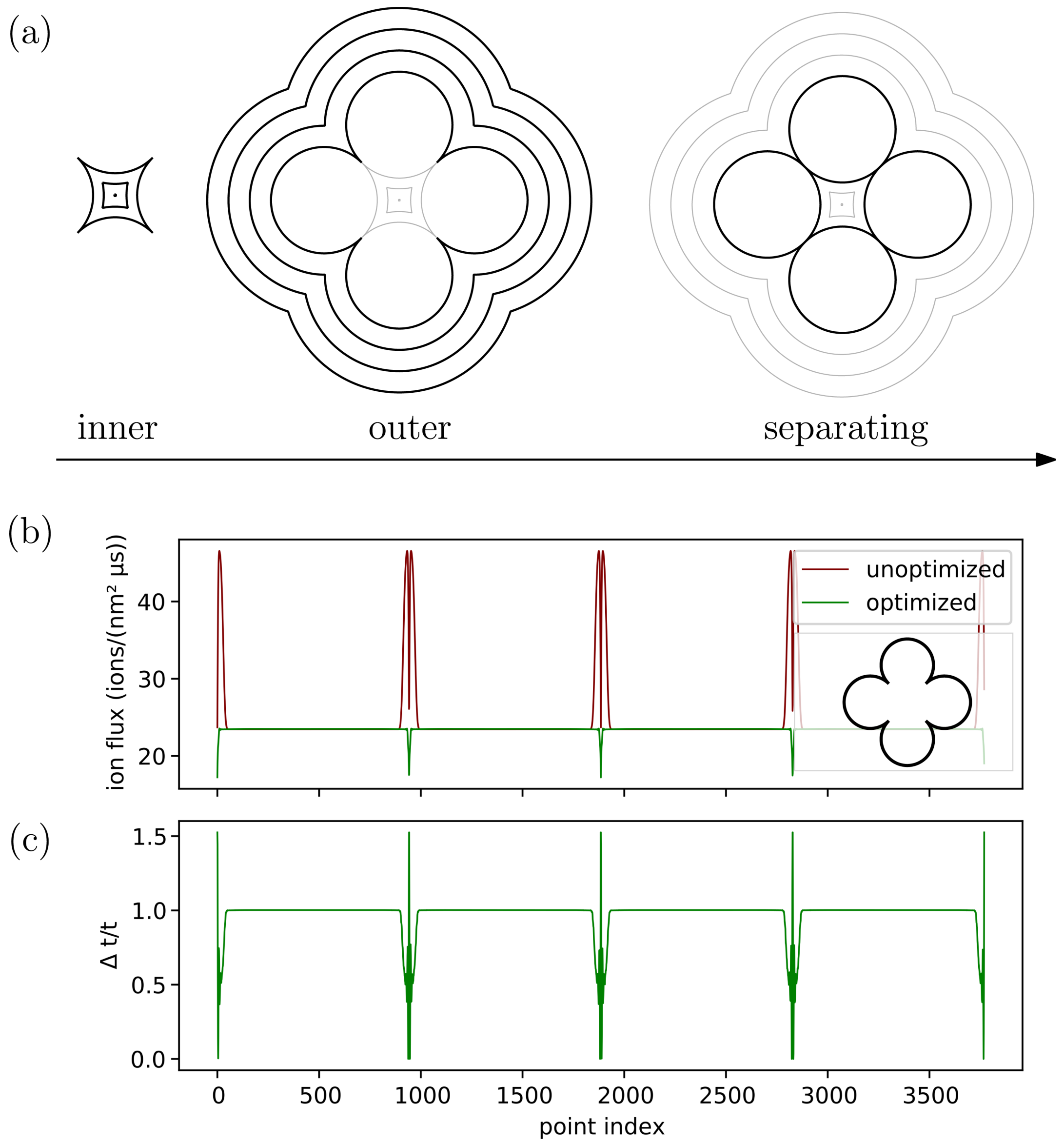

Figure S5: Optimization routine for the tetramer pattern.

Figure S5a shows the structure of the tetramer pattern. First, the inner part is milled (pitch = 0.5 nm, offset pitch = 0.25 nm, two repeats with alternating direction). Second, the outer part is patterned (pitch = 0.25 nm, offset pitch = 0.25 nm, two repeats with alternating direction). Finally, two "8"-shaped separating cuts are made (pitch = 0.25 nm, 50 repeats). The global base dwell time is 5 μs and the complete sequence is repeated for 55 times. Figure S5b depicts the ion flux for each point on a curve before and after local dose optimization. After the optimization step, the local doses are alomost homogeneously distributed. Only the innermost points (cf. the curve shown in the inset)

have lower doses because these points are less influenced by surrounding points and need much higher dwell times than all other points to get the same ion flux. This is also visible in Figure S5c where the relative change in the dwell time per point is plotted. The innermost points exhibit a much higher relative dwell time. The maximum relative dwell time is set to 1.5. In doing so, no artifacts caused by high local doses are introduced.

As mentioned in the main manuscript, heat may not be the only source of the beam-induced damage observed in the tetramers. Redeposition may appear in addition to the melting of structures. Hence, further systematic studies are required. Here, we report an observation that may point towards the influence of local heat creation. Figure S3b–e shows tetramers with different types of deformations such as material agglomeration towards the center of the tetramer, where the largest local dose was applied (Figure S3b), the displacement of separate monomers in the tetramer (Figure S3c), and a shift/movement of gold in non-patterned regions at a rim of the gold flake (Figure S3d) that did not occur for smaller local doses (Figure S3e). The material agglomeration in Figure S3b could also be redeposited material. Only a TEM investigation on a cross section could elucidate this. However, the separate displacement of monomers in S3c is hard to explain without taking heat into account. The material shift in S3d may also result from a more efficient material removal by secondary ions at the edge. Also here, further systematic investigations, ideally accompanied by molecular dynamics simulations (coupled to a heat transport equation), would be required for a definite conclusion. Further, it is important note that for all these effects contamination played a critical role since it increased the minimum dose that is required to remove the material. Interestingly, we never observed this type of deformations for sputtered gold on a silicon test sample, which we used for the adjustment of the microscope and the first patterning tests.

References

1. FIB-o-mat documentation. https://fib-o-mat.readthedocs.io/en/latest/ (accessed Nov 09, 2020).

2. FIB-o-mat gitlab repository. https://gitlab.com/viggge/fib-o-mat/ (accessed Nov 09, 2020).

3. Buffat, P.; Borel, J. J.-P. *Phys. Rev. A* **1976**, *13*, 2287. doi:10.1103/PhysRevA.13.2287.

4. Granqvist, C. G.; Buhrman, R. A. *J. Catal.* **1976**, *42*, 477–479. doi:10.1016/0021-9517(76)90125-1.

5. Cybenko, G. *J. Parallel Distrib. Comput.* **1989**, *7*, 279–301. doi:10.1016/0743-7315(89)90021-X.
